# Evaluation of Spinal Alignment and Clinical Findings for the Efficacy of One-Stage Surgery in Tandem Spinal Stenosis

**DOI:** 10.7759/cureus.25130

**Published:** 2022-05-19

**Authors:** Tatsuki Kobayashi, Eguchi Yawara, Munetaka Suzuki, Takashi Sato, Masaya Mizutani, Hajime Yamanaka, Hiroshi Tamai, Sumihisa Orita, Kazuhide Inage, Yasuhiro Shiga, Satoshi Maki, Junichi Nakamura, Shigeo Hagiwara, Yasuchika Aoki, Masahiro Inoue, Masao Koda, Hiroshi Takahashi, Tsutomu Akazawa, Seiji Ohtori

**Affiliations:** 1 Department of Orthopaedic Surgery, Shimoshizu National Hospital, Yotsukaido, JPN; 2 Department of Orthopaedic Surgery, Graduate School of Medicine, Chiba University, Chiba, JPN; 3 Department of Orthopaedic Surgery, Eastern Chiba Medical Center, Togane, JPN; 4 Department of Orthopaedic Surgery, University of Tsukuba, Tsukuba, JPN; 5 Department of Orthopaedic Surgery, St. Marianna University School of Medicine, Kawasaki, JPN

**Keywords:** cervical spinal stenosis, clinical symptoms, spinal alignments, lumbar spinal stenosis, tandem spinal stenosis

## Abstract

Introduction

We compared preoperative skeletal muscle, pre-and post-operative spinal alignment or clinical symptoms between tandem spinal stenosis (TSS) patients who underwent simultaneous cervical and lumbar decompression and lumbar spinal stenosis (LSS) patients who underwent only lumbar decompression and the efficacy of one-stage surgery was examined.

Methods

This study included 82 patients, identifying 13 patients for the TSS group (mean age 77.2 years) and 69 patients for the LSS group (mean age 72.2 years). One-stage decompression surgery was performed on both groups. The spinal alignments were evaluated using Lumbar scoliosis (LS), Sagittal vertical axis (SVA), Lumbar lordosis (LL), Pelvic tilt (PT), Pelvic incidence (PI), and Sacral slope (SS). The clinical symptoms were evaluated using the visual analogue scale (VAS) score for low back pain (LBP), the Japanese Orthopedic Association scoring system (JOA score), the Roland-Morris Disability Questionnaire (RDQ), the Japanese Orthopedic Association Back Pain Evaluation Questionnaire (JOABPEQ) and Oswestry Disability Index (ODI).

Results

The amount of bleeding was not significantly different between the two groups (p > .05). SVA, LL, PT, and SS were significantly improved in the LSS group (p < 0.05). In the TSS group, SVA, LL, PT, and SS tended to improve, but without significant differences. The proportion of JOABPEQ gait dysfunction that was difficult to climb stairs was 83% in the TSS group, and social life disturbance that was difficult to engage in ordinary activities was 67% in the TSS group, which was significantly higher than that in the LSS group (p < .05). Although clinical symptoms improved by surgery in both groups (p < .05), there was no significant difference in the degree of clinical symptom improvement before and after surgery (p > .05).

Conclusions

One-stage surgery for TSS is effective because it has the same intraoperative bleeding volume as LSS alone and is minimally invasive. It also improves forward-leaning posture and clinical symptoms equivalent to LSS alone.

## Introduction

With society aging, the number of patients with spinal stenosis increases annually. Spinal stenosis often is caused by age-related changes such as disc degeneration and osteophyte formation, which are frequently found in the highly mobile cervical and lumbar spine [1-3]. The prevalence of cervical spondylotic myelopathy (CSM) is estimated to be 5% to 20%, and lumbar spinal stenosis (LSS) is estimated to be 8% to 11% [4,5]. However, it has been reported that stenosis is observed at a considerably higher rate on simple X-rays, including in asymptomatic elderly people [2,3] Clinical symptoms differ depending on whether the stenosis site is outside or in the center of the spinal canal, or in the intervertebral foramen [6]. Tandem spinal stenosis (TSS) is a disease in which stenosis is observed in both the cervical spine and the lumbar spine and can present with variable symptoms [7].

Conservative treatment or surgical treatment is selected according to the symptoms [1,8], but there is still no consensus about whether surgical treatment should be a one-stage approach or a two-stage approach [9]. In addition, there are reports of improved alignment after surgery for LSS [10], but no reports for TSS.

The purpose of this study was to compare preoperative spinal alignment, skeletal muscle mass, clinical symptoms, and surgical results between TSS patients who underwent simultaneous cervical and lumbar decompression and LSS patients who underwent only lumbar decompression.

## Materials and methods

Participants

Informed consent was obtained from all participants before the study began. The study protocol was approved by the Shimoshizu National Hospital Ethics Review Committee (No. of IRB: H26'-6).

From December 2015 to May 2021, 82 patients were identified who visited our outpatient department of orthopedics, were diagnosed with LSS or TSS, received surgical treatment, and were followed for more than six months and an average of three years. Surgery was selected when the patient had difficulty in daily activities due to neuropathy caused by TSS or LSS. Of the 82 cases (mean age 72.5 years, range 34-86 years, 38 men), 13 cases (mean age 77.2 years, range 66-86 years, 2 men) were diagnosed with TSS, and 69 cases (mean age 72.2 years, range 34-84years, 36 men) were diagnosed with LSS.

In the TSS group, there were three patients who had 1-level cervical decompression, seven with 2-level decompression, and three with 3-level decompression. There were six who had 1-level lumbar decompression, six with 2-level decompression, and one with 3-level decompression. The cervical levels decompressed in the TSS group were: C3/4: 5 cases, C4/5: 9 cases, C5/6: 9 cases, and C6/7: 1 case; and in the lumbar spine Th12/L1: 1 case, L1/2: 1 case, L2/3: 4 cases, L3/4: 9 cases, L4/5: 6 cases, and L5/S: 1 case. The number of laminectomies was 3.6.

In the LSS group, there were 51 1-level lumbar decompressions, 11 2-level decompressions, four 3-level decompressions, and two 4-level decompressions. Levels decompressed in the LSS group were Th12/L1: 1 case, L1/2: 1 case, L2/3: 5 cases, L3/4: 21 cases, L4/5: 43 cases, and L5/S: 11 cases. The number of laminectomies was 1.4. The TSS group had significantly more laminectomies than the LSS group (p < 0.05). Lumbar spinous process-splitting laminectomy (L-SPSL) [11] was performed for all LSS cases.

For TSS cases, myovascular preserving open-door laminoplasty with mini-plate fixation (MPLP) [12] was performed on the cervical spine. Patients were positioned prone with their heads secured using Mayfield clamps. Surgery for the TSS group was performed on the same day by the same surgeon (Y.E.) in the order of MPLP for the cervical spine and L-SPSL for the lumbar spine. Patients with spinal tumors, infectious diseases, or spinal trauma were excluded.

Radiographic analysis 

The anterior and lateral views of the lumbar spine were photographed with patients in a standing position. Radiographic measurements were made of lumbar scoliosis (LS), the sagittal vertical axis (SVA), lumbar lordosis (LL), pelvic tilt (PT), pelvic incidence (PI), and sacral slope (SS). The LS was measured from anterior radiographs as the angle between the lower end plate of L1 and the lower end plate of L5. The SVA was measured on lateral radiographs as the distance from the C7 plumb line to a perpendicular line drawn from the superior posterior end plate of the S1 vertebral body. The LL was measured from the lower end plate of T12 to the upper end plate of S1. The PT was measured as the angle between the vertical line and the line joining the hip axis with the center of the superior end plate of S1. The PI was measured as the angle subtended by a perpendicular line from the upper end plate of S1 and a line connecting the center of the femoral head to the center of the cephalad end plate of S1. The SS was measured as the angle between the superior end plate of S1 and a horizontal line. 

Analysis of skeletal muscle mass and phase angle

A multifrequency bioelectrical impedance analyzer (BIA; MC-780A, TANITA, Tokyo, Japan) was used according to the manufacturer’s guidelines. BIA is a noninvasive examination technique used for evaluating bone mass, fat mass, and fat-free mass by flowing weak currents at three different frequencies (5, 50, and 250 kHz) using eight electrodes in total, two for each sole and grip in standing barefoot position and determining the difference in electric resistance. The analysis time is less than 20s. Limb and trunk muscle masses were determined directly from the value of lean mass provided by the device. 

Appendicular skeletal muscle mass was calculated as the sum of skeletal muscle mass in the arms and legs, assuming that the mass of lean soft tissue is effectively equivalent to skeletal muscle mass. The appendicular skeletal mass index (SMI) was determined as the sum of arm and leg lean mass (kg)/(height (m)^2^.

Resistance (R) and reactance (Xc) measured at 50 kHz were used for the calculation of the phase angle by: Phase angle (degrees) = arc-tangent (Xc/R) × (180°/π).

Clinical symptoms

Clinical symptoms were evaluated using the visual analogue scale (VAS) score for low back pain (LBP), leg pain, and leg numbness ranging from 100 mm (extreme amount of pain) to 0 mm (no pain); the Japanese Orthopedic Association (JOA; 0-29 points) scoring system; and the Roland-Morris Disability Questionnaire (RDQ; 0-24 points). The normal JOA score is 29 points, based on three subjective symptoms (9 points), three clinical signs including straight-leg raising (6 points), and seven activities of daily living (14 points). The normal RDQ is zero points with the total number of items checked from a minimum of 0 to a maximum of 24.

The Japanese Orthopedic Association Back Pain Evaluation Questionnaire (JOABPEQ) includes 25 questions based on RDQs and Short Form 36 (SF-36). For Q1-1 through Q4-1 and Q5-1, a score of “1” was considered positive for symptoms, while “2” or “3” was considered negative. For Q4-2, Q4-3, and Q5-2 through Q5-7, a score of “1” or “2” was considered positive for symptoms, and “3” to “5” were considered negative. Scores are calculated based on the answers to questions in 5 domains: pain-related disorders, lumbar spine dysfunction, gait disturbance, social life dysfunction, and psychological disorders. The score for each domain was calculated according to the official guidelines and ranged from 0 to 100 points, which is deemed proportional to the patient’s clinical condition.

Statistical methods

Measurements were made of surgery time, intraoperative bleeding volume, radiographic measurements, age, height, weight, body mass index (BMI), body fat percentage, skeletal muscle mass, phase angle, and clinical symptoms for all participants in both groups. For each variable and clinical symptom, differences between groups were evaluated using an unpaired t-test. For each JOABPEQ item, differences between both groups were evaluated using the chi-squared test. All data are expressed as the mean ± standard deviation (SD). A value of p < 0.05 was considered significant. Statistical analyses were performed using Statistic Analysis System (SAS) for Windows (Version 9.4, SAS Institute Inc., Cary, NC).

## Results

There were no significant differences in patient age, height, weight, BMI, or body fat percentage between the two groups (p > 0.05), nor were there any differences between groups in skeletal muscle mass (Table 1). 

**Table 1:** Intraoperative findings, physical examination, skeletal muscle mass, and phase angle in the two groups TSS: Tandem spinal stenosis; LSS: Lumbar spinal stenosis

	TSS	LSS	p-value
Surgery			
Time (minutes)	159.30±31.9	88.10±40.7	p<0.05
Bleeding volume (ml)	103.40±30.7	68.20±55.0	0.076
Level of decompression			
Cervical decompression			
1 level	3		
2 level	7		
3 level	3		
Lumbar decompression			
1 level	6	51	
2 level	6	11	
3 level	1	4	
4 level		2	
Mean No. of decompression	3.6	1.4	p<0.05
Physical examination			
Age (yrs)	77.2±6.3	72.2±9.6	0.273
Height (cm)	154. 7±9.3	159.7±8.0	0.152
Weight (kg)	60.3±13.7	61.5±9.77	0.791
BMI (kg/m^２^)	24.9±4.1	24.0±2.8	0.476
Body fat percentage (%)	28.9±7.6	25.3±7.2	0.232
Lean body muscle (kg)	40.2±8.8	43.5±8.41	0.360
Truncal muscle (kg)	22.1±4.1	24.6±3.91	0.123
Upper limbs muscle (kg)	4.2±1.2	4.4±1.2	0.520
Lower limbs muscle (kg)	13.8±4.0	14.4±3.7	0.656
SMI (kg/m^２^)	7.4±1.5	7.3±1.4	0.777
Phase angle			
H-L (left half body)	-5.2±0.8	-6.7±1.3	0.005
RL (right lower limbs)	-4.1±0.9	-5.4±0.9	0.002
LL (left lower body)	-4.1±1.0	-5.4±0.9	0.001
RH (right hand)	-6.7±0.9	-7.3±0.8	0.065
LH (left hand)	-6.2±0.8	-7.8±1.5	0.012
L-L (both lower limbs )	-4.0±1.0	-5.4±0.9	0.001

**Figure 1:**
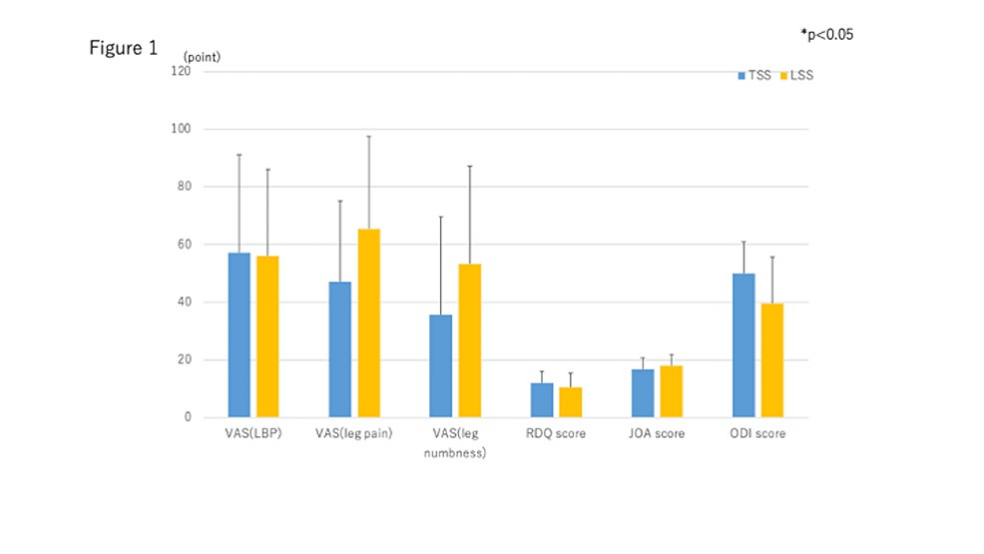
Preoperative clinical symptoms in both groups Preoperative clinical symptoms in both groups There was no significant difference in preoperative clinical symptoms between the two groups. VAS, Visual Analogue Scale; JOA, Japanese Orthopaedic Association; RDQ, Roland-Morris Disability Questionnaire; ODI, Oswestry Disability Index; TSS, Tandem spinal stenosis; LSS, Lumbar spinal canal stenosis.

There were no significant differences in preoperative clinical symptoms between the two groups (Figure 1). 

Phase angle of the left half body, left hand, and both lower limbs were significantly lower in TSS than in LSS (p < 0.05, Table 1). The operation time (min) was 159.3 ± 31.9 in the TSS group and 88.1 ± 40.7 in the LSS group (p < 0.001). No significant differences were found between groups in the amount of intraoperative bleeding (Table 1).

**Figure 2:**
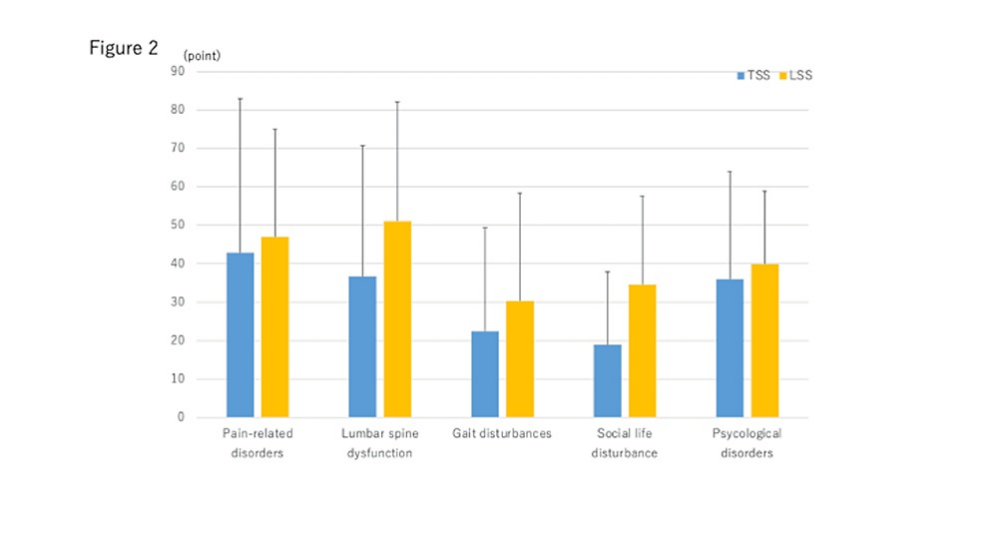
Preoperative JOABPEQ scores in each group Preoperative JOABPEQ scores in each group There was no significant difference between the two groups in any JOABPEQ category. JOABPEQ, Japanese Orthopaedic Association Back Pain Evaluation Questionnaire; TSS, Tandem spinal stenosis; LSS, Lumbar spinal canal stenosis.

Categories in JOABPEQ include pain-related disorders, lumbar spine dysfunction, gait disturbances, social life disturbances and psychological disorders. There were no significant differences between the two groups in any of these categories of the JOABPEQ (Figure 2). 

However, statistically significant differences were noted between the two groups for difficulty climbing stairs (p = 0.007) and in the ability to engage in ordinary activities (p = 0.040) (Table 2).

**Table 2:** Percentage of cases in the two groups reporting symptoms based on the JOABPEQ

	Questionnaire	TSS(%)	LSS(%)	p-value
Q1-1	Change posture often	60.0	65.9	0.311
Q1-2	Lie down to rest	50.0	31.7	0.239
Q1-3	Lower backaches	40.0	46.8	0.519
Q1-4	Cannot sleep well	0	29.3	0.678
Q2-1	Require help with tasks	31.3	24.4	0.519
Q2-2	Avoid bending forward or kneeling	40.0	53.7	0.605
Q2-3	Have difficulty standing up from a chair	33.3	34.1	0.605
Q2-4	Have difficulty turning over in bed	33.3	46.3	0.967
Q2-5	Have difficulty putting on socks	66.7	43.9	0.204
Q2-6	Have difficulty: bending forward, kneeling, or stooping	33.3	17.1	0.353
Q3-1	Can only walk short distances	50.0	70.1	0.688
Q3-2	Stay seated most of the day	50.0	51.2	0.678
Q3-3	Go up the stairs more slowly than usual	83.3	73.2	0.470
Q3-4	★Have difficulty climbing stairs	83.3	14.6	0.007
Q3-5	Cannot walk more than 15 minutes	66.7	39.0	0.199
Q4-1	Cannot do housework	66.7	17.1	0.134
Q4-2	★Cannot engage in ordinary activities	66.7	22.0	0.040
Q4-3	Work routine is hindered	60.0	43.9	0.094
Q5-1	Feel irritated	14.3	17.1	0.176
Q5-2	Poor health condition	71.4	70.7	0.581
Q5-3	Feel depressed or discouraged	28.6	17.1	0.805
Q5-4	Feel exhausted	28.6	22.0	0.739
Q5-5	Do not feel happy	14.3	39.0	0.186
Q5-6	Am not in decent health	57.1	36.6	0.910
Q5-7	Worry health will get worse	42.9	43.9	0.375

**Figure 3:**
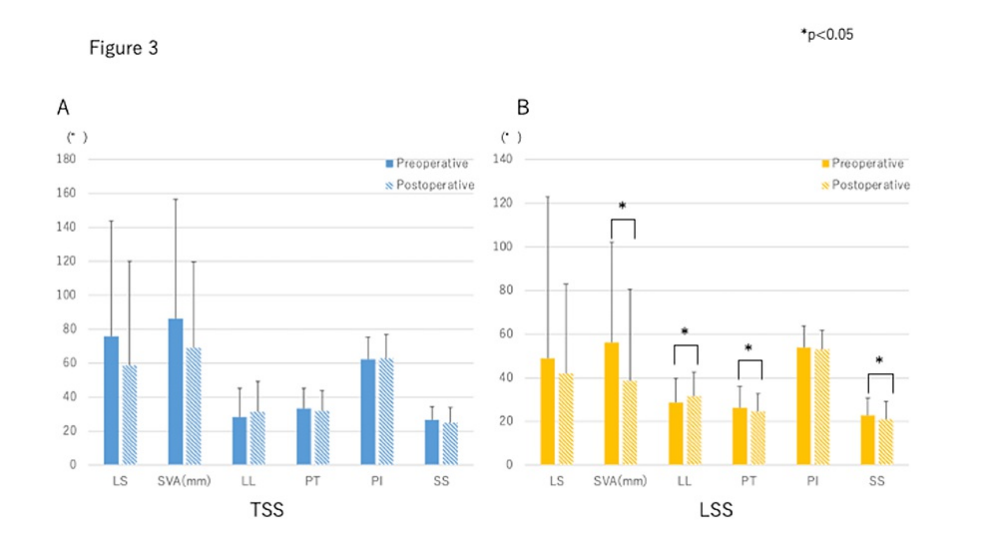
Pre-and postoperative spinal alignment in both groups Preoperative and postoperative spinal alignment in both groups Postoperatively, SVA, LL, PT and SS showed a tendency to improve, but there was no significant difference with preoperative values. In the LSS group, SVA, LL, PT, and SS were improved significantly after surgery (p < 0.05). Index; TSS, Tandem spinal stenosis; LSS, Lumbar spinal canal stenosis; LS, Lumbar scoliosis; SVA, Sagittal vertical axis; LL, Lumbar lordosis; PT, Pelvic tilt; PI, Pelvic incidence; SS, Sacral slope.

There were no significant preoperative to postoperative differences in spinal alignment for TSS patients (Figure 3).

Postoperatively, SVA, LL, PT, and SS showed a tendency to improve, but without significant differences. On the other hand, in LSS patients, SVA (p = 0.04), LL (p = 0.01), PT (p = 0.01), and SS (p = 0.03) all improved significantly after surgery (Figure 3).

**Figure 4:**
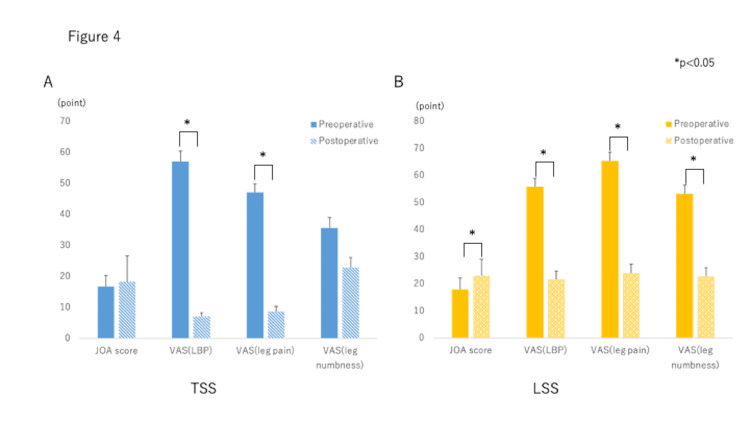
Pre-and postoperative clinical symptoms in both groups Pre- and post-operative clinical symptoms in both groups VAS (LBP) and VAS (leg pain) were significantly improved after surgery in the TSS group (p < 0.05). On the other hand, the JOA score, VAS (LBP), VAS (leg pain) and VAS (leg numbness) all were improved significantly in the LSS group after surgery (p < 0.05). There were no significant differences between the groups in the degree of clinical symptom improvement.

Postoperatively in the TSS group, the VAS (LBP, p = 0.005) and VAS (leg pain, p = 0.014) scores improved significantly. On the other hand, the JOA score (p = 0.00007), VAS (LBP) (p = 0.000002), VAS (leg pain) (p = 0.0000002) and VAS (leg numbness) (p = 0.00008) scores all improved significantly after surgery in the LSS group (Figure 4). 

However, there were no significant differences in the degree of clinical symptom improvement (postoperative-preoperative) by decompression between the two groups. 

## Discussion

TSS was first reported by Teng and Papatheodorou in 1964 [7]. Dagi et al. helped to further define TSS by three clinical signs: (1) intermittent lower extremity claudication, (2) mixed motor neuron signs of upper and lower limbs, and (3) progressive gait disturbance [13]. The prevalence is reported to be high in women, ranging from 7.6% to 60% of patients with spinal stenosis [7,8,13-16]. Kawaguchi et al. have reported an association between posterior longitudinal ligament ossification (OPLL) and TSS [14], but the causes and risk factors for TSS are not yet clear. 

The prevalence of TSS varies in different studies. Lee et al. [4] and Bajwa et al. [17] reported a prevalence of TSS of 2-5.4% in two different studies using 1072 and 440 cadavers, respectively. Kawaguchi et al. reported that the prevalence of TSS was 19-60% when there was stenosis in either the cervical or lumbar region, using diagnostic imaging such as simple X-rays and CT [14]. Early suspicion of TSS is important because TSS often presents with residual symptoms and is frequently diagnosed after surgery on either the cervical or lumbar spine. Lee et al. showed that lumbar spinal stenosis symptoms at multiple levels in men over 70 years old is a risk factor for TSS [4]. Iizuka et al. reported that the only other independent risk factor for TSS is a cervical Torg-Pavlov ratio (the ratio between the developmental sagittal diameter and the vertebral body diameter at the same level from cervical spine radiographs) of < 0.78 [16]. The Torg-Pavlov ratio was reported to be significantly lower in patients with cervical spondylotic myelopathy [18,19].

In this study, we compared spinal alignment, skeletal muscle mass, clinical symptoms, and surgical results in the TSS group with those in the LSS group. There were no significant differences in skeletal muscle mass between the two groups, but the trunk muscle mass and phase angle (left half body, both lower limbs, and left hand) were significantly lower in the TSS group than in the LSS group.

Phase angle (PhA) is high in normal cells with a structurally complete cell membrane, such as in healthy subjects and athletes, and low in cells with structural damage to the cell membrane, such as occurs with aging and in cancer patients. Clinically used as an index for assessing nutritional status, low PhA is said to be significantly associated with nutritional risk, length of hospital stay, and mortality [20,21]. In recent years, the relationship between PhA and skeletal muscle mass also has attracted attention. In this study, PhA in the left half of the body and both lower limbs was significantly lower in the TSS group, suggesting that aging of the cell membranes of the trunk and both lower limbs was progressive. In lumbar spinal stenosis, multi-level stenosis is more prone to severe neuropathy than single intervertebral stenosis which occurs more cranially. It has been suggested that spinal nerve compression in both the cervical and lumbar spine exacerbates the senescence of the trunk and both lower limb skeletal muscle cells distal to the stenosis [2]. Detailed research on the relationship between PhA and spinal disorders is needed in the future.

There were no significant differences in preoperative clinical symptoms such as VAS score, RDQ, JOA score, and Oswestry Disability Index (ODI) score between the two groups. These assessments were primarily disease-specific assessments for low back pain and may not reflect the effects of cervical myelopathy in TSS patients.

Nearly 85% of TSS cases were characterized by difficulty in climbing stairs and about 67% had difficulty engaging in ordinary social activities, consistent with the mixed symptoms and signs of the upper and lower limbs. In the TSS group, "difficulty climbing stairs" improved from 85% to 33%, and "difficulty engaging in ordinary activities" improved from 57% to 0% after surgery. The proportion of patients who found it difficult to climb stairs was higher in the TSS group than in the LSS group, consistent with the symptoms of TSS characterized by progressive gait disturbance [13]. If there is difficulty in climbing stairs or engaging in ordinary activities that do not match the severity of LSS, it is necessary to treat TSS with cervical myelopathy in mind.

RDQ and ODI are used as specific scales of low-back-pain-associated quality of life, while SF-36 and EuroQol are widely used internationally as comprehensive measures of health. The JOABPEQ is a patient-based evaluation of treatment results that includes both scientific and psychological assessments [22]. An excel file can be shared from the JOA website, allowing for automatic assessment of individual patient severity. Based on this questionnaire, Eguchi et al. reported that resting leg pain, such as difficulty in wearing socks, is a characteristic of lumbar foraminal stenosis in LSS patients [23]. If the JOABPEQ shows resting pain or intermittent claudication, further diagnosis and treatment should consider the possibility of LSS [23]. 

Hsieh et al. showed that elimination of lumbar symptoms did not necessarily require tandem decompression in patients with symptomatic TSS [8]. Additionally, Epstein et al. found that all patients treated with primary cervical decompression had improvement in their lower extremity symptoms [1]. Eskander et al. [24] suggested that age greater than 68 years, estimated blood loss over 400 mL, and surgical time over 150 minutes should prompt the clinician to consider staged rather than single-setting surgery. Compared to the two-stage approach, the one-stage approach has a shorter surgical time and less bleeding, so the burden on the patient is reduced. In addition, the one-stage approach can prevent complications such as pressure on the cervical nerves when the patient is in the lumbar surgical position. However, there was no difference in clinical results between the two surgical approaches [9,12,24,25]. In this study, one-stage surgery was performed on all TSS cases by a single surgical team. The amount of intraoperative bleeding was equivalent to that of LSS alone, and significant improvement in clinical symptoms was observed, suggesting that the one-term approach to TSS is effective.

Since the MPLP method is a minimally invasive surgery that preserves the muscles of the cervical spine and has less bleeding with an average of 100 ml [12], it is considered that there was no significant difference in the amount of bleeding between the two groups even if the cervical spine surgery was added.

Krishnan et al. reported that the cervical and lumbar spine were operated on by different teams at the same time, and the operation time was 150 minutes, slightly less than the 159 min. reported here for tandem decompression. That method might be useful [25].

We performed only decompression on both the cervical and lumbar spine. In the systematic reviews of Overley et al. [9] and the reports of Eskander et al. [24], only decompression was performed, but other studies report that fusion surgery is added if there is instability [10,26,27].

Schwab et al. investigated the correlation between health-related quality of life (ODI) and vertebral pelvic parameters in 492 patients with adult spinal deformity and found that they correlate with PT, SVA, PI, and LL [28]. Glassman et al. reported that SVA is associated with clinical symptoms [29]. Ogura et al. reported that SVA was improved by decompression alone for LSS without instrumentation because the patient leans forward to relieve symptoms [30]. However, there have been no reports about TSS and spinal alignment. In this study, decompression in the LSS group significantly improved SVA, LL, PT, SS, and clinical symptoms. On the other hand, in the TSS group, SVA, LL, PT, and SS, were not significantly different but tended to improve. Thus, decompression surgery improved spinal alignment and clinical symptoms in both groups, suggesting that one-stage decompression surgery for TSS also may be indicated.

Our study has several limitations. First, a small number of subjects were investigated, requiring confirmation of our findings in a larger population. Second, the TSS group was slightly older than the LSS group, although not significantly older. Nevertheless, the TSS group may have had some age-related loss of skeletal muscle mass. Third, this study did not measure cervical alignment such as C2-7 SVA, C2-7 angle. Changes in cervical alignment should be further investigated by one-stage surgery for TSS. Finally, one-stage surgery and two-stage surgery in the TSS group were not compared.

## Conclusions

This study showed that one-stage surgery for TSS is effective because it has the same intraoperative bleeding volume as LSS alone and is minimally invasive. It also improves forward leaning posture and clinical symptoms equivalent to LSS alone.
